# Perspectives on Modifying Attentional Biases Amongst Individuals with Tobacco Use Disorder Using Technology: A Review

**DOI:** 10.3390/ijerph16152644

**Published:** 2019-07-24

**Authors:** Yi Zhuang Tan, Melvyn W.B. Zhang, Carol C. Choo

**Affiliations:** 1 Department of Psychology, College of Healthcare Sciences, James Cook University, Singapore 387380, Singapore; 2 Family Medicine and Primary Care, Lee Kong Chian School of Medicine, Nanyang Technological University, Singapore 308232, Singapore

**Keywords:** attentional bias, attentional bias modification, smoking, mobile app, technology

## Abstract

Smoking remains a significant health problem. Attentional biases influence smoking behaviours, but have not been the target of psychosocial interventions. The first part of this perspective article will provide an overview of the theoretical constructs underlying attentional biases, methods of measuring attentional biases, and evidence for attentional bias modification amongst individuals with tobacco use disorders. The second part of this article will outline how the advent of technological advances could be harnessed in attentional bias modification for smokers. As there is potential for attentional bias training to be delivered via mobile app, literature was reviewed over the recent decade, 2009 to 2019, to examine available research evidence. The search terms were “web-based” or “mobile based”, and “attention bias modification” or “attentional bias” and “smoking” or “tobacco use”. The PsycINFO, Scopus, and PubMed databases were initially used to identify papers with the above-mentioned inclusion criteria. Five papers were included in the review. Lastly, an integrated perspective will be provided, from both clinical and research standpoints. In conclusion, more research is needed to address the gaps in knowledge and to provide an evidence base for the implementation of mobile phone technologies for attention retraining in smokers.

## 1. Introduction

The World Health Organization has highlighted that smoking remains a current global health threat [1]. Smoking is associated significantly with mortality, with figures estimated to be seven million deaths yearly [1]. Smoking leads to mortality not only for individuals who smoke, but also for passive smokers who are exposed to second-hand smoke, with 890,000 deaths resultant from second-hand passive smoking. Smoking is especially prevalent in low- and middle-income countries [1]. Given the extent of the problem, there is a variety of policy and regulatory changes in place to help address this major public health issue [1]. Public education campaigns are conducted, in order to raise the awareness of smoking and its relationship with other medical comorbidities [1]. For individuals who are keen to quit, medications, psychosocial interventions, and counseling are common approaches used [1]. Pharmacological measures include nicotine replacement therapy and medications such as bupropion and varenicline [2]. Psychosocial treatment involves behavioural support at individual and group levels [2]. Psychological interventions informed by the stages of change model [3,4], as well as therapeutic techniques from motivational interviewing [5], cognitive behavioural approaches [6], and self-management strategies [5], hold promise to change problematic behaviours [4,7]. Regulatory changes that are in place include having graphic pack warnings on tobacco products, limiting tobacco advertisements, as well as increasing taxes on tobacco products [1]. 

### 1.1. Attentional Biases amongst Tobacco Users

Cigarettes are the most commonly used tobacco products, with tobacco use disorders prevalent among individuals who use cigarettes daily [8]. Attentional biases are unconscious processes whereby individuals with tobacco use disorder will focus automatically on smoking-related cues in their environment [9]. Although these processes influence behaviours, they have not been the targets of most common psychosocial interventions [3,4,5,7]. In addition to the conventional psychosocial interventions mentioned, understanding of attentional biases in smoking could inform effective and innovative strategies that target these automatic processes. The first part of this perspective article will provide an overview of the theoretical constructs underlying attentional biases, methods of measuring attentional biases, and evidence for attentional bias modification amongst individuals with tobacco use disorders. The second part of this perspective article will provide a brief overview of how the recent advent of technological advances could be harnessed in attentional bias modification for these individuals and the effectiveness of such technologies. Lastly, an integrated perspective will be provided, from both clinical and research standpoints.

#### 1.1.1. Theoretical Constructs

Several theoretical constructs have posited that attentional biases are present and responsible for slips and relapses in individuals with tobacco use disorder. The incentive-sensitization theory [10] highlighted that substance-related cues could acquire incentive-motivational properties. Addiction (e.g., cigarette smoking) is caused primarily by substance-induced sensitization in the mesocorticolimbic systems that attribute salience to reward-associated cues [11]. Repeated substance consumption results in increased dopaminergic response that causes the substance to be perceived as salient in the brain, and it becomes a goal to be pursued, which increases the subjective craving in cigarette smoking. Repeated pairing of cues, specifically the sight of cigarettes to the dopaminergic response from smoking could condition the cue to acquire similar motivational properties as the actual act of smoking. Thus, these cues become more salient in the environment, and capture smokers’ attention easily. Incentive sensitization produces biased attentional processing towards substance-related cues and increases craving towards the substance [10]. Franken [12] extends this theory by suggesting that there is a bi-directional relationship between attentional bias for substance-related cues and subjective craving for the substance. As dopamine directs one’s attention to stimuli that predict or signal reward [13] through classical conditioning, substance-related stimuli become more salient, which increases subjective craving. Following this increased craving, attentional bias towards the substance is further increased, which consolidates the whole cycle of craving and attentional bias in the addiction. The elaborated intrusion (EI) theory of desires makes similar predictions about the reciprocal relationship between attentional bias towards substance-related cues and subjective craving towards the substance. Kavanagh, Andrade, and May [13] posited that cognitive elaboration on the substance of desire is the key component perpetuating cravings, which makes an act of consumption more likely. Subjective craving is initially experienced as an intrusive thought, being triggered by an external cue, such as the sight of a cigarette [14], which drives the individual to elaborate on it cognitively. This increases attentional allocation to substance-related cues, which strengthens the craving [15].

Lastly, the theory of current concerns [16] proposes a relationship between attentional biases towards cue-related substance and subjective craving. Current concern is the state of an individual between becoming committed to pursuing a particular goal and attaining the goal or giving up the pursuit [16]. Commitment to a goal pursuit triggers a latent brain process that sensitizes an individual to respond emotionally and to take notice, recall, think about, and act on cues associated with the goal pursuit. The valence of the goals may be either positive (i.e., approach goals) or negative (i.e., avoidance goals). An approach goal for a smoker will be smoking, in order to achieve the dopaminergic response from the nicotine consumption, while an avoidance goal will be smoking to counteract the withdrawal symptoms of abstinence from smoking. With the motivational state biasing a smoker’s cognitive process towards goal-related stimuli, the smoker will inevitably pay more attention to such stimuli. With this increased attention towards substance-related stimuli, this theory ties in with prior theories in supporting how attentional bias towards substance-related stimuli increases craving and motivation towards consuming the substance, which increases the likelihood of consumption and perpetuating the cycle by sensitizing dopaminergic response. 

In summary, whilst the theories mentioned above have suggested different mechanisms in which attentional bias develops, they ultimately converge on the same idea that attentional bias and subjective craving have a bi-directional causal relationship with each other.

#### 1.1.2. Measures of Attentional Biases

The Stroop task and visual-probe task are common reaction-time based tasks used for the measurement of attentional biases. Attentional bias in these tasks is usually measured indirectly from impaired performance in a task due to attention being drawn to substance-related stimuli. One of the most common measures of substance-related attentional bias is a modification of the Stroop task. In the original Stroop task, often considered the gold standard of attention measures [17], subjects are instructed to state the colour of the ink in which a series of colour words are printed in [18]. The time taken for subjects to name the colour accurately is inversely proportional to their ability to suppress the semantic of the word stimulus. In the modified Stroop task to study addiction, often referred to as the addiction Stroop task, subjects are presented with words related to their addiction, such as ‘lighter’, which is a smoking-related word; and ‘paper’, which is a neutral word. In addiction studies, attentional bias, measured in duration of time, is inferred from the potency of the distractors, i.e., from the impairment of subjects’ ability to name the colour of a word stimulus when the stimulus is a substance-related word. 

Several studies utilizing the Stroop task with smokers have provided evidence of smokers’ attentional bias towards smoking-related stimuli [19,20,21]. These studies found that smoking-related stimuli interfered with smokers’ performance in the Stroop task, which suggests attention being drawn to such stimuli, thus impairing performance [20]. However, alternative mechanisms may also explain the impaired performance. Firstly, smokers may be slower to name the colour of smoking-related word stimuli as such stimuli may produce cognitive states that interfere with the ability to name the colour efficiently [22]. Smoking-related stimuli could trigger motivational states that increase subjective craving, which utilizes cognitive resources [23]. Due to the increased cognitive load, smokers’ performance on smoking-related stimuli suffers. Secondly, impaired performance on smoking-related stimuli could be due to smokers’ attempts to suppress intrusive thoughts about smoking. Smokers who are currently abstaining from smoking may be actively trying to avoid thoughts related to smoking, which could contribute to impaired performance on substance-related stimuli [24]. As there are no avoidance response options in the Stroop task, the impairment could be ascribed to mood-congruent response bias, where subjects’ motivational factors influence response, rather than attentional bias [25]. 

Overall, although the Stroop task is a well-established attentional measure, when modified for addiction studies, we must be cautious and wary in drawing the same interpretation and association of attention theory underlying the results. The hallmark of the original Stroop task is the separation of word stimuli into congruent (e.g., the word ‘red’ displayed in red colour) and congruent groups (e.g., the word ‘red’ displayed in blue colour), and this difference is where attentional effect is drawn [26]. In contrast, congruence does not play a major role (e.g., smoke in red colour is no more congruent than road in red colour) in addiction Stroop task. Instead, the effect difference is more emotionally and motivationally driven, thus different mechanisms could explain the results drawn.

The visual-probe task is an alternative to the Stroop task. This task [27] requires subjects to respond to the location of a dot probe by pressing a key as quickly and accurately as they can. Prior to the dot probe being displayed, subjects are shown two images side-by-side on a computer screen. Quicker reaction times when a dot probe replaces a particular category of images suggest biased attention to that category. Two categories of images are shown (e.g., images related to smoking versus neutral images). Attentional bias is measured by the difference of the scores between the reactions times to dot probes following one category and reaction times to dot probes following the other. Several studies have utilized the visual-probe task in studies of smoking, and expectedly yielded results that showed smokers displayed attentional bias, by attending differentially to smoking images [28,29]. There are a few advantages of the visual-probe task over Stroop task as a measure of attentional bias towards smoking-related stimuli. Firstly, the visual-probe task requires subjects to make a neutral response (i.e., button press) to a neutral stimulus (dot probe), which minimizes any response bias due to the motivational state of the subject [26]. Secondly, the visual-probe task more accurately reflects real-life scenarios that a smoker faces. This is because the visual-probe task requires subjects to split attention between two different stimuli as compared to only focusing on one stimulus at a time in the Stroop task. In the real world, smokers constantly have to split their attention between different stimuli, thus the visual probe task can better capture this aspect, which allows results to be more generalizable.

#### 1.1.3. Attentional Bias Modification

Whilst there have been major advances in experimental psychology in the last decade, the field of attentional re-training in smoking is relatively new, with limited number of studies done. However, it holds potential clinical utility as an adjunct tool to smoking interventions. As mentioned above, various theories of attentional bias have indicated that attentional bias results from the repeated pairing of cues such as the sight of cigarettes to the dopaminergic response from smoking, which leads to sensitized reaction to such cues and thus they become more salient. The saliency perpetuates the vicious cycle as it increases attention to smoking cues, which is considered relevant to smoking cessation outcomes [29]. In contrast to smokers who have increased attentional bias towards smoking-related cues, former smokers showed avoidance towards such cues [30]. Thus, attentional bias modification in smokers may be crucial in contributing to a higher rate of smoking cessation success. 

The first attempt at attentional re-training in smokers utilized a modified version of the visual-probe task. The modified version utilized the same paradigm as the original visual-probe task, with the only difference being that the dot probe always replaced the neutral stimuli [31]. In other words, subjects were trained to prioritize their attention towards the neutral stimuli over the smoking-related stimuli. The results demonstrated that following attention bias modification, there was a significant decrease in post-training attentional biases towards smoking-related stimuli. Despite this initial positive finding, subsequent studies using such single-session training on smokers showed mixed results, and have not been able to consistently replicate the results [32,33,34].

Other researchers have investigated the effectiveness of bias modification by increasing the frequency of attentional re-training. Lopes and colleagues [35] were the first to employ multiple-session trainings in a longitudinal study. In their study, they randomly allocated subjects enrolled in a smoking cessation program into one of three groups: three sessions of attentional bias training; two sessions of placebo training (visual probe task with neutral pictures) with one session of attentional bias training; and three sessions of placebo training. All three groups exhibited decreased attentional bias towards smoking-related stimuli 24 hours after training. At one-month after training, subjects from two groups (three sessions of attentional bias training and two sessions of placebo training with one session of attentional bias training) exhibited decreased attentional bias. Interestingly, at 6 months, subjects from the purely attentional bias training group still maintained this effect. These results indicated that multiple sessions of attentional bias training could produce more robust changes in attentional bias towards smoking-related stimuli.

### 1.2. Technological Advances and Attentional Bias Re-Training

Whilst Lopes and colleagues [35] demonstrated that bias modification was more robust by increasing the frequency of attentional bias re-training, it is challenging in the real world to get subjects to commit to multiple sessions over a long period of time, due to the inconvenience it places on their personal schedules. An efficient way to curtail this difficulty will be to conduct attentional bias re-training over mobile devices, given their benefits for high-dosage treatment delivery, and prevalent usage and convenience. Additionally, it was suggested that electronic interventions delivered via mobile phone apps could enhance patient-centered care in an increasingly technology-savvy society, underscoring the importance of interventions delivered using electronic platforms [36,37,38]. Smartphone apps have been used to deliver psychological therapies such as addiction treatment [39]. In view of the barriers to seeking help from counselors and psychologists, which might include stigma, time and financial constraints, there might be preference to access community interventions such as self-help on electronic platforms delivered using smartphone apps, rather than face-to-face therapy [37]. Apps offer an alternative delivery medium that is also easily accessible, convenient and cost effective. Delivering mental health treatment through mobile devices hold several advantages, as it is ubiquitous, and enables people in remote regions with limited access to mental health services to have greater opportunities to obtain treatment [36,40].

## 2. Methods

In view of the reasons stated above, there is potential for attentional bias training to be delivered via mobile app, thus literature was reviewed over the recent decade, 2009 to 2019, to examine the available research evidence. The database search was conducted in March 2019, using these search terms: “web-based” or “mobile based”, and “attention bias modification” or “attentional bias” and “smoking” or “tobacco use”. 

## 3. Results

**Figure 1:**
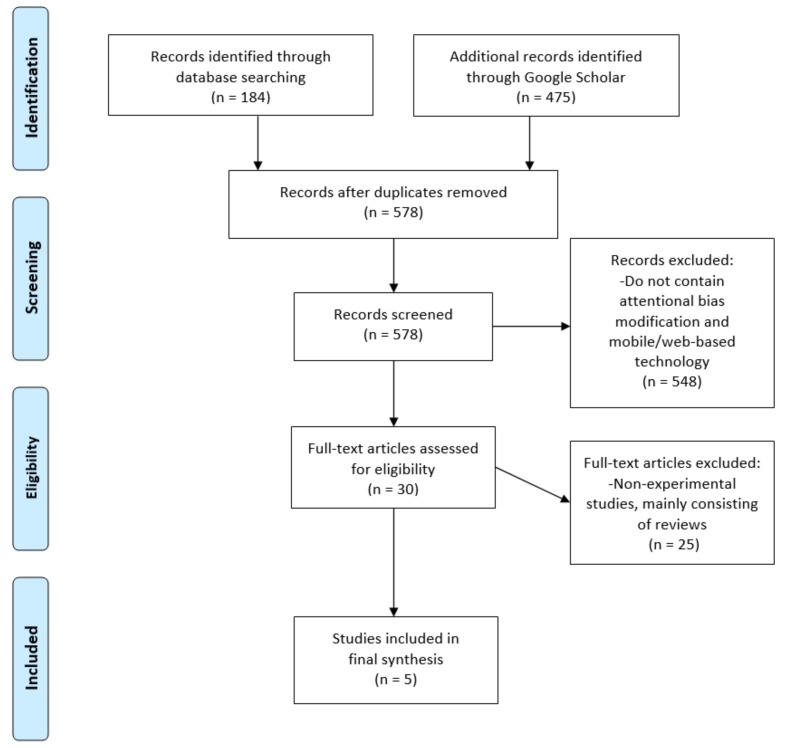
PRISMA (Preferred Reporting Items for Systematic Reviews and Meta Analyses) flow diagram.

The PsycINFO, Scopus, and PubMed databases were initially used to identify peer-reviewed papers with the inclusion criteria named above, which yielded 184 results, using all search terms. Additional records were identified through Google Scholar and yielded 475 additional results. From the original search results, 81 duplicated articles were removed, and 578 abstracts were screened; 30 full text papers from peer-reviewed journals were then downloaded and assessed against the inclusion and exclusion criteria. The structured pro forma for evaluating eligibility for inclusion involved the following: recent papers that contain original experimental studies published in peer-reviewed journals after the year 2009; original research related to usage of web-based or mobile based, attentional bias modification for smokers. The focus was on recently published papers in peer-reviewed journals that fit the inclusion criteria. The main reason for the exclusion of articles was that papers were not original experimental studies. Two researchers were responsible for the evaluation and selection of the studies included in the final review. Any cases of discrepancies were considered, and a consensus was reached with the research team, which included a third researcher. Figure 1 illustrates the review process. 

The results are presented in Table 1 below, and provide an overview of the studies identified. Five papers were included in the final review. The evidence was mixed for web-based attentional bias modification interventions. Wittekind et al. [41] and Elfeddali et al. [42] evaluated the efficacy of online web-based intervention in large samples of smokers. Wittekind et al. [41] reported a reduction in the number of cigarettes smoked and smoking compulsion in participants, whereas Elfeddali et al. [42] reported that attentional bias modification (ABM) training had no significant effect regarding bias reduction and no behavioral effects in the whole sample of smokers, and subsample analyses revealed a significant positive effect on continued abstinence in heavy smokers only. In addition, Wittekind et al. [43] found that although the approach bias modification-training group demonstrated reduced daily consumption of cigarettes immediately after training, as compared with a wait list control, no differences were observed at the six-month follow-up period. 

For interventions delivered via a mobile device, two American studies were found. Kerst and Waters [44] were the first to have utilized personal digital assistants (PDAs) to deliver attentional re-training (AR) to smokers. Participants were placed into two groups: AR group in which they underwent three modified visual probe tasks for training and one standard visual probe task for assessment daily; or control group in which they underwent three standard visual probe tasks for training and one standard visual probe task for assessment daily. Participants were required to commit for a week and could do the tasks at their own convenience whenever the PDA prompted them to. Results found that attentional bias towards smoking-related stimuli and subjective craving decreased over the week for the AR group. Robinson et al. [45] examined the effect of attentional re-training, which was delivered via PDA devices to participants who were non-treatment seeking African American smokers. The results demonstrated the effectiveness of attentional bias re-training, as there was significant reduction in attentional biases to smoking cues, and this was generalized to new images.

## 4. Discussion

The advent of technology has ushered in growing interest in harnessing the use of technology to deliver interventions in recent years. The review of available literature in Table 1 shows preliminary but mixed evidence that mobile devices could be harnessed in the delivery of attentional bias modification intervention for individuals with smoking problem. Gaps remain, specifically the application of mobile devices was limited to Personal Digital Assistant [44,45]. There has been an absence of available research on rigorous evaluation of a smartphone app for modification of attentional biases in smoking, which underscores the importance of future research into this innovative area. 

Previous literature highlighted the importance of interventions delivered using smartphone apps, in view of the potential to reduce health care costs in Asia [37]. In comparison to Western countries, there is a shortage of mental health professionals in Asia, yet a high penetration of mobile phone usage throughout Asia, with Singapore alone reporting that smartphone adoption rates far exceeded the population. In addition, the studies reviewed in Table 1 were conducted in Western countries [42,43,44,45], and generalizability to Asian countries is unclear. Recent literature had cautioned against the assumption that research outcomes from Western countries could be generalized to Asian populations, due to cultural differences [46,47,48], therefore studies in Asian populations are important in order to inform the delivery of ethno-culturally sensitive health services in Asia [47]. In addition, there is suggestion that cultural disparities exist in acceptance and usage of tobacco, due to income, education and tobacco control in different countries [8]. However, such important sociocultural factors were largely unexplored in the studies reviewed. One of the studies reviewed did not consider demographic information in the main analysis [41], and there was only a brief mention in another study conducted in African Americans [45]. None of the studies reviewed were specifically conducted in Asian countries. There remains a need for future research in establishing the potential of smartphone apps in the modification of these underlying biases; and their overall effectiveness for Asian countries. In addition, smoking is especially prevalent in low- and middle-income countries [1], and in developing countries [8], however, the studies reviewed were conducted in developed countries, and generalizability is limited for developing nations and low- and middle-income countries. Furthermore, this review included American studies [44,45] and European studies [42,43], it is unclear if findings could be extended to Australia and New Zealand, and if outcomes might differ between European and indigenous smokers in Australia [36] and New Zealand.

Tobacco use disorders are characterized by craving or strong urge to use tobacco, as well as continued and recurrent use of large amounts of tobacco over lengthy period, leading to clinically significant impairment [8]. The review of available literature in Table 1 suggests mixed evidence that mobile devices delivering attentional bias modification (ABM) intervention could be used effectively for tobacco use disorders. Research found significant reduction in attention bias [44,45], craving [44], compulsion [41], and number of cigarettes smoked [41,43] among participants who received intervention versus control [41]. However one study found that ABM training had no significant effect regarding bias reduction and no behavioral effects in the whole sample of smokers [42]. The reduction in attention bias did not consistently correspond to a reduction in craving or biological measures of smoking or cigarettes smoked [45]. In addition, the outcomes were not maintained at six-month follow up [43]. As tobacco use is associated with chronic diseases such as cardiovascular diseases, chronic obstructive pulmonary diseases, cancers and co-morbid psychiatric conditions [8], it is crucial for an effective intervention to yield sustained outcomes to reduce risk for these adverse conditions.

Considering the prevalence of smoking problems, and the adverse health outcomes [1], research is much needed to explore alternative ways to deliver effective interventions. Previous research has indicated that apps show promise for use with low-income and ethnically diverse populations, to help with coping and facilitate recovery [36], and could be harnessed to offer a convenient platform to deliver attentional re-training for smokers. However, previous research has cautioned that it is crucial to appreciate that any treatment or therapy might have the potential for harm, and that any device might cause adverse effects with incorrect usage. Some critiques have focused on the ethical responsibility to protect consumers from potential harm. The argument follows that if apps were used by consumers for self-help therapy, it is important that the app stores are reputable and that the apps are created by legitimate third-party software developers. Principles of rigorous scientific inquiry should be applied to explore the sustained benefits of the use of such health-related apps. Future research ought to verify the evidence base for commercialized bias modification apps. With the growing popularity of e-interventions, it is expected that there will be increasing availability of such commercialized apps. Identification of the evidence base of smoking bias modification is essential prior to the commercialization of such apps, given that some individuals who wish to seek help with smoking, might attempt to use such apps as a form of self-help therapy, instead of seeking face-to-face therapy. It is essential for there to be scientific scrutiny, and guidelines as to which health- related apps are recommended and which are not. From a clinical perspective, it is essential to examine the effectiveness of such innovative e-interventions in promoting abstinence and maintenance of abstinence. The critical issue for health care professionals and clinicians when recommending such mobile phone apps as an adjunct tool, to their patients, to supplement face-to-face therapy or as stand-alone self-help therapy, is the risk to benefit ratio. Apps should be rigorously assessed for effectiveness with the target population. Additionally, a critique has been made on whether patients could become dependent on apps. However, problem smokers are not a homogeneous group, and this must be considered when clinicians are recommending on app suitability for their patients. 

## 5. Conclusions

In summary, there is consensus that smoking continues to be a widespread problem with adverse consequences. This perspective article provided an overview of the theoretical constructs and evidence for attentional bias modification (ABM) amongst individuals with tobacco use disorders, and considered how technology could be harnessed to deliver ABM. As mobile delivery of interventions aimed at attention re-training holds promise to support people who are seeking to change their smoking behavior, literature was reviewed over the recent decade, 2009 to 2019, to examine available research evidence. The search terms were “web-based” or “mobile based”, and “attention bias modification” or “attentional bias” and “smoking” or “tobacco use”. In comparison to the theoretical background and relevant literature suggesting the potential clinical utility for apps designed for attention re-training interventions for smokers, the current review reveals a relative paucity on the evidence base available for consistent and sustained outcomes for mobile delivery of interventions aimed at attention re-training for tobacco use disorders.

The current review has identified only five relevant papers on the topic, and underscores that future research is needed on this important and innovative topic. The evidence base has been examined and discussed for individuals with tobacco use disorders, but the review reveals mixed results in consistent and sustained benefits for smokers with clinically significant impairment. More research is needed to address the gaps in knowledge and to provide rigorous evidence base for sustained benefits of mobile phone technologies in attention re-training for tobacco use disorders. Cultural influences should also be considered—American and European studies were reviewed but generalizability to Asian countries is unclear. Furthermore, the evidence base is insufficient for comprehensive understanding of sustained benefits of such apps for developing countries, middle and low-income populations, and Indigenous as well as culturally diverse groups. Developing mobile tools for consumers with smoking problems requires careful ethical consideration regarding overall best practice guidelines. More rigorous research and evaluations are needed to ascertain the efficacy of and establish evidence for best practice for use of such mobile phone apps with consumers who are seeking help for smoking problems. Quality and ethical issues relating to the use of mobile apps in attention re-training in smokers need to be considered on a deeper level, before such apps are commercialized, either as a stand-alone self-help tool or as an adjunct tool to supplement face-to-face therapy. Cautious clinical judgment should be applied when considering usage with smokers suffering from tobacco use disorders. 

## Figures and Tables

**Table 1:** Summary of evidence.

Author/Year	Description of Intervention	Outcomes
Wittekind et al., (2015) [41]	Web-based Approach - Avoidance Task (AAT)	Bias present and subjected to manipulation. Significant reduction in number of cigarettes smoked *F* = 3.55, *p* = 032 and compulsion *F* = 3.32, *p* = 0.039 among participants who received intervention versus control. Reduction of cigarette dependence and compulsive drive for smoking most significant in those assigned to the standard Approach - Avoidance Task (AAT).
	N = 257 smokers (Demographic information not specified)	
Elfeddali et al., (2016) [42]	Web-based Attentional Bias Modification, ABM training (Visual Probe task)	The ABM training had no significant effect regarding bias reduction and no behavioral effects in the whole sample of smokers (p>.15). Subsample analyses revealed a significant positive effect on continued abstinence in heavy smokers only, *OR* = 3.15; *p* = 0.02. ABM effects did not generalize to that of approach bias.
	Approach bias using reaction time paradigms	
	N = 434 Dutch adults	
Kerst & Waters (2014) [44]	Attentional Retraining (AR) via Personal Digital Assistants (PDAs)	Reduction in attentional bias *PE* = 31.4, *p* = 0.01, *d* = 0.69, and overall craving for smoking *PE* = 0.77, *p* = 0.04.
	N = 60 adult smokers in United States of America (USA)	
Robinson et al., (2017) [45]	Attentional Retraining (AR) via Personal Digital Assistants (PDAs)	Attentional biases were reduced in AR group versus control, *F* = 9.20, *p* = 003. The reduction in attention bias did not correspond to a reduction in craving or biological measures of smoking/ cigarettes smoked.
	N= 64 African American adults	
Wittekind, Lüdecke and Cludius, (2019) [43]	Web-based approach-bias modification	Approach bias modification (ABM) training group demonstrated reduced daily consumption of cigarettes immediately after training compared with a wait list control, *t*(32) = 2.89, p = 0.007, but no differences were observed at the 6-month follow-up period.
	N = 149 German adults	No consistent change in bias emerged through training, and no support for it being a stand-alone intervention for smoking.
